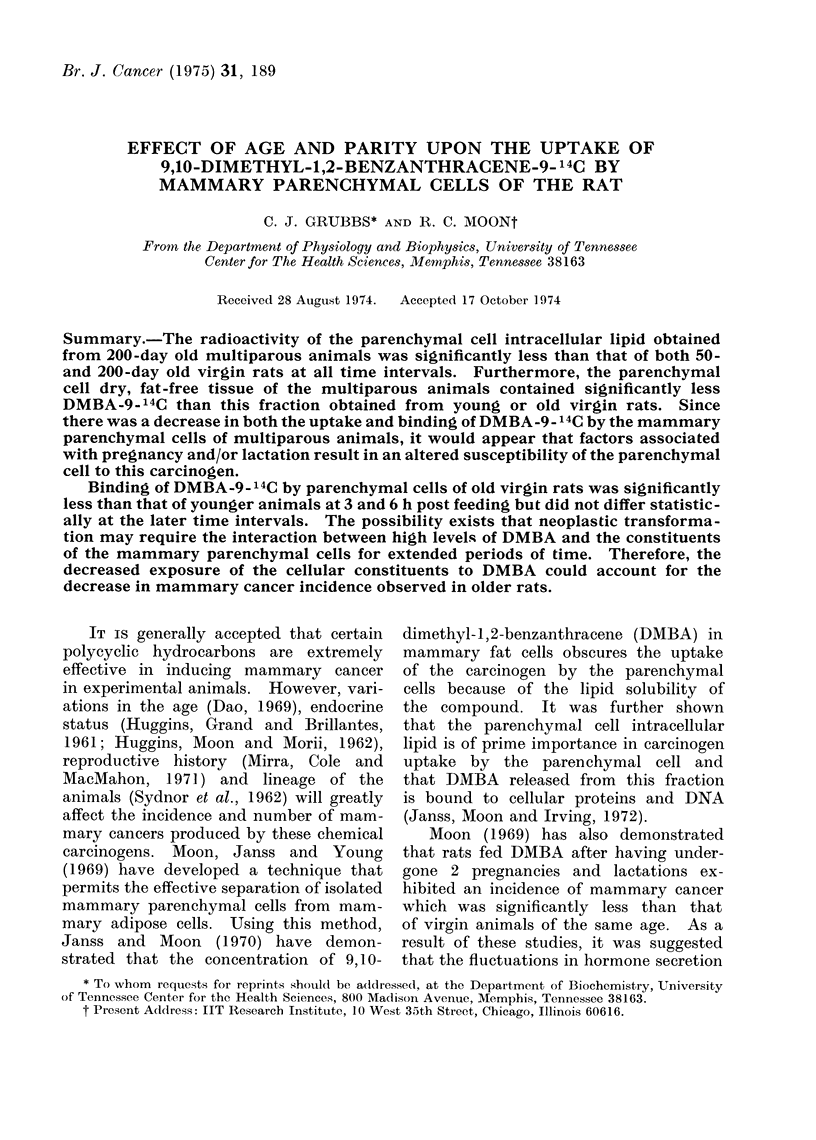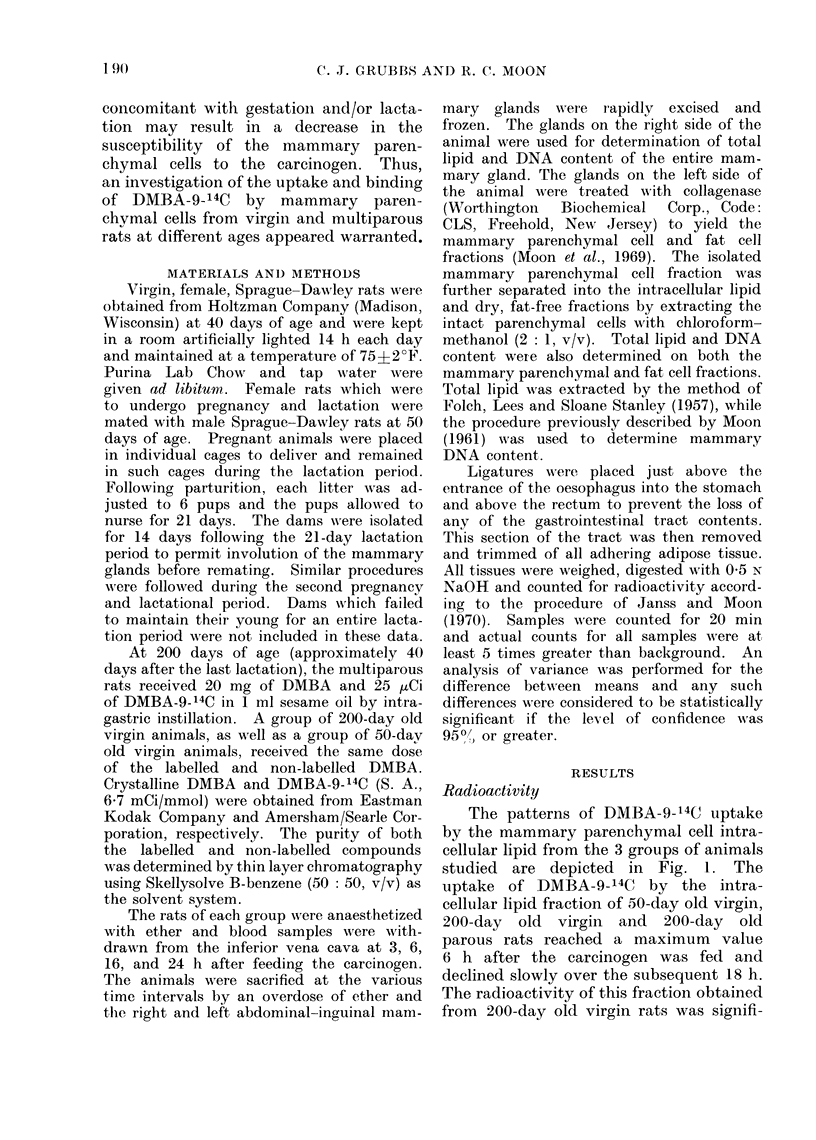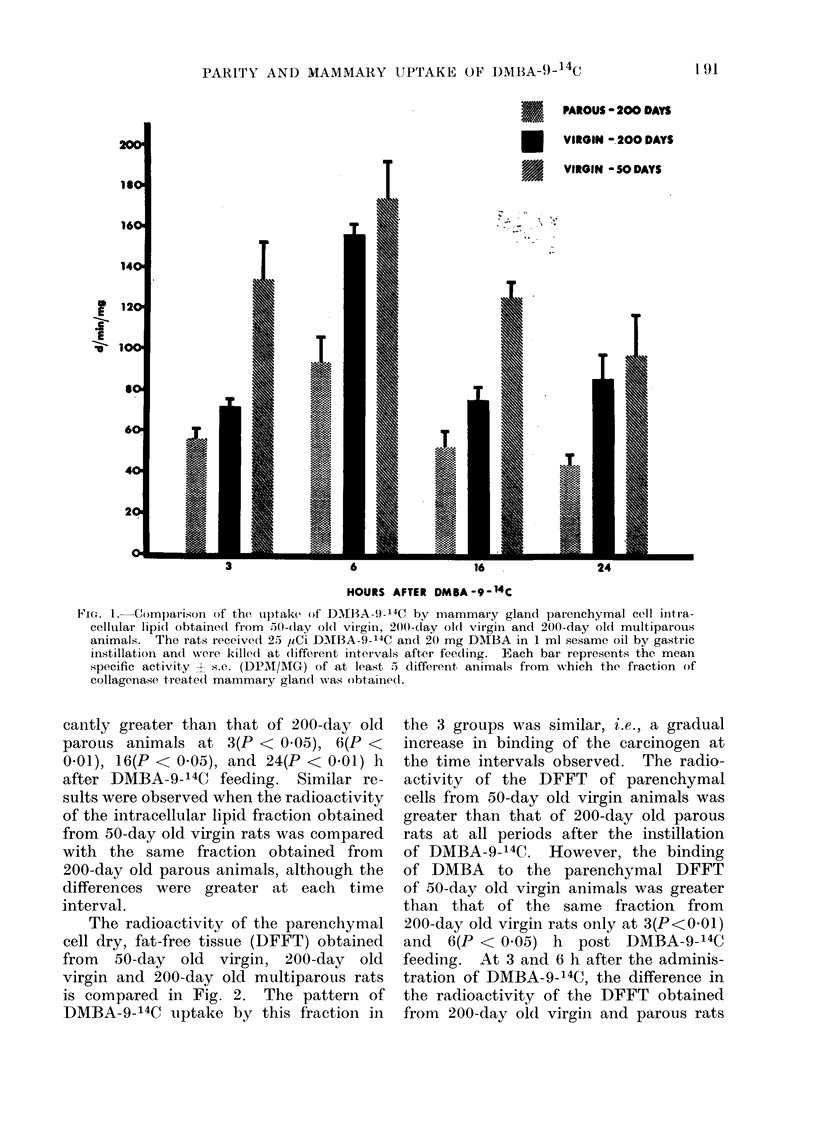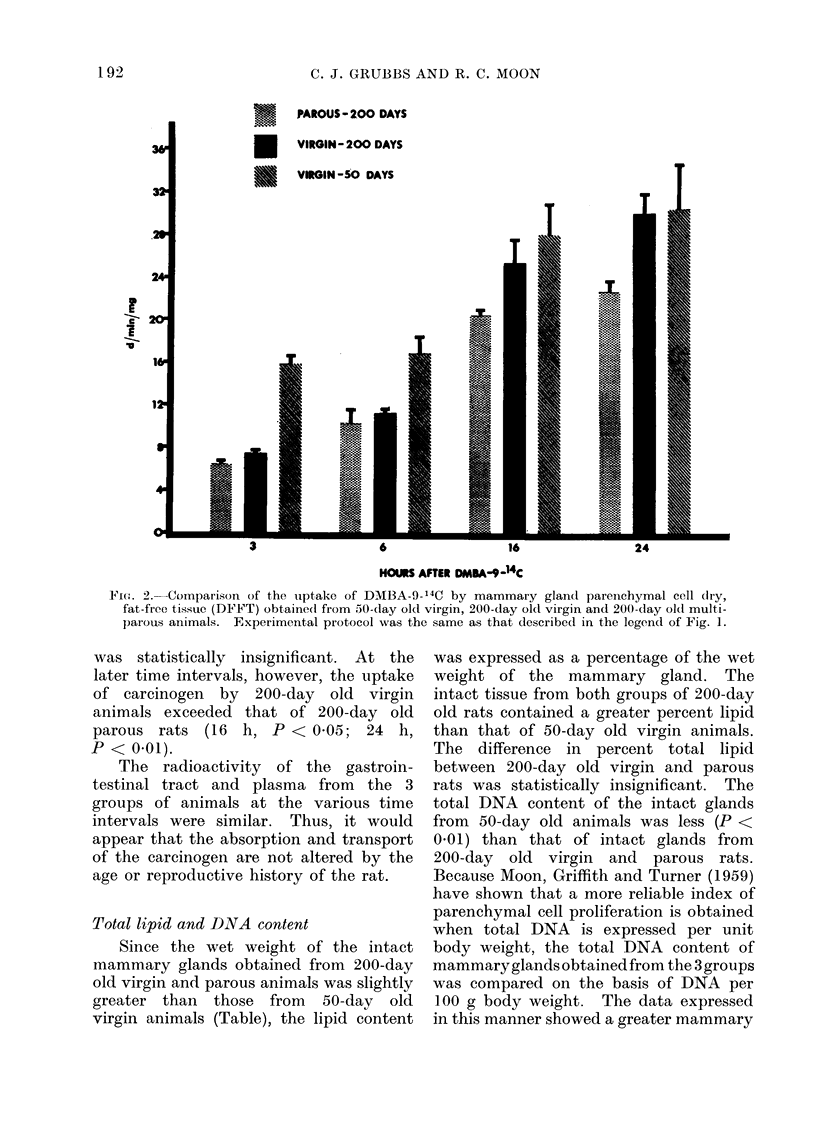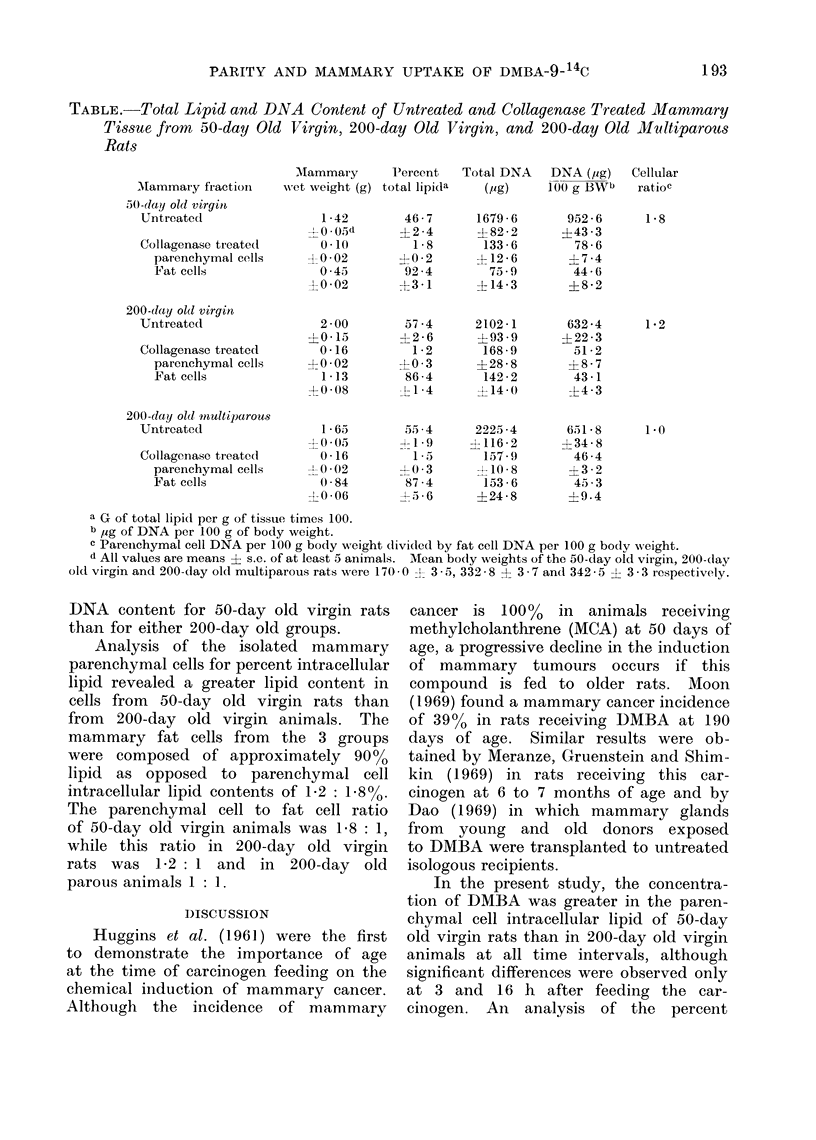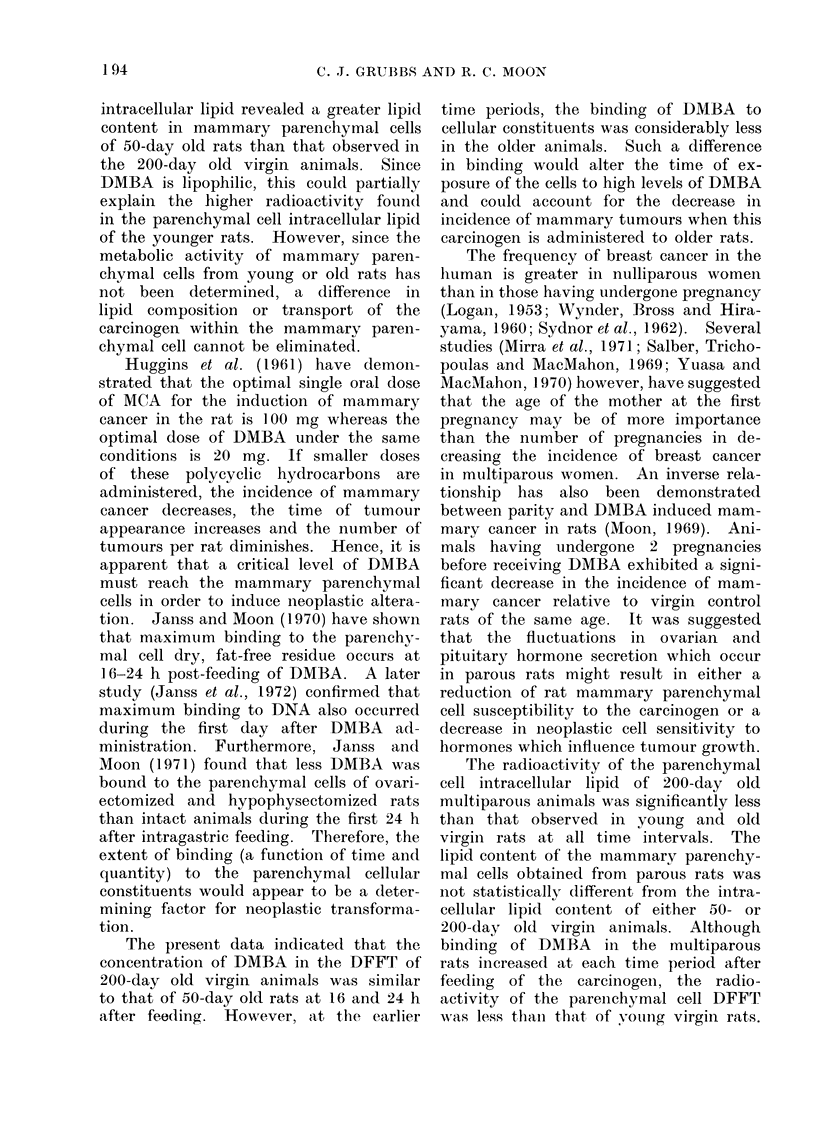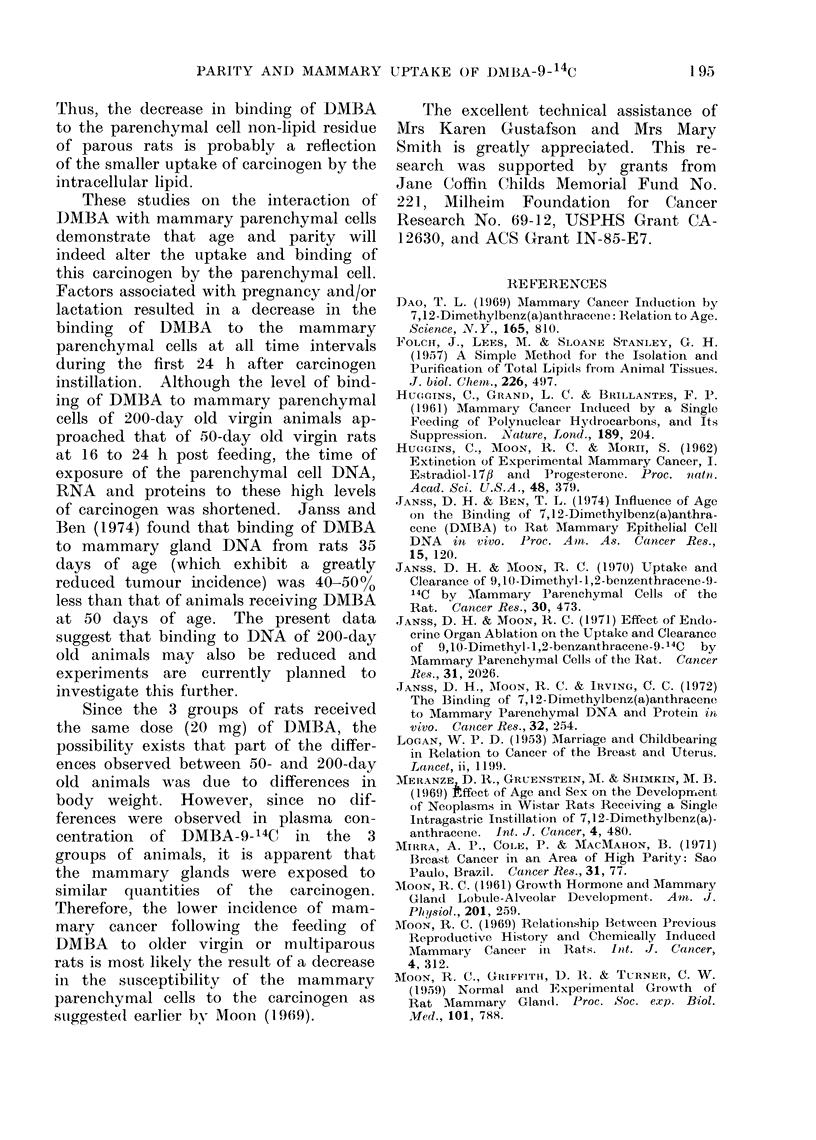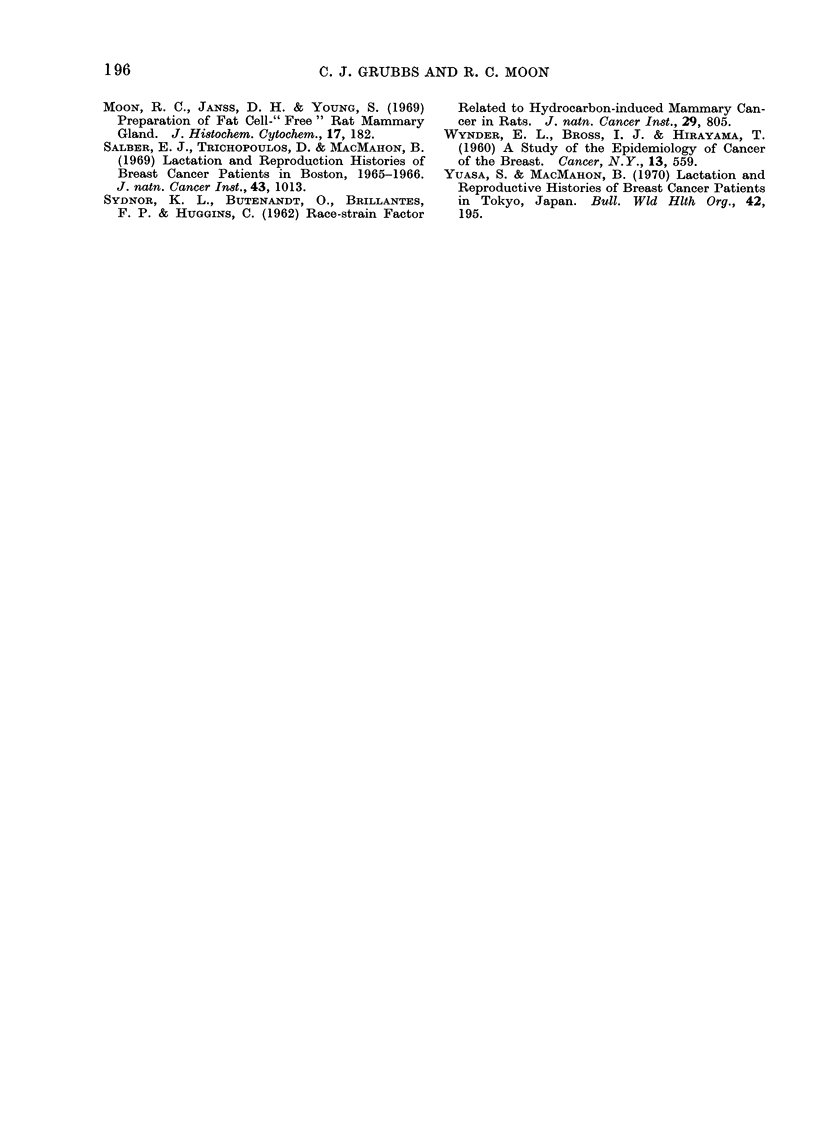# Effect of age and parity upon the uptake of 9,10-dimethyl-1,2-benzanthracene-9-14C by mammary parenchymal cells of the rat.

**DOI:** 10.1038/bjc.1975.25

**Published:** 1975-02

**Authors:** C. J. Grubbs, R. C. Moon

## Abstract

The radioactivity of the parenchymal cell intracellular lipid obtained from 200-day old multiparous animals was significantly less than that of both 50- and 200-day old virgin rats at all time intervals. Furthermore, the parenchymal cell dry, fat-free tissue of the multiparous animals ocntained significantly less DMBA-9-14C than this fraction obtained from young or old virgin rats. Since there was a decrease in both the uptake and binding of DMBA-9-14C by the mammary parenchymal cells of multiparous animals, it would appear that factors associated with pregnancy and/or lactation result in an altered susceptibility of the parenchymal cell to this carcinogen. Binding of DMBA-9-14C by parenchymal cells of old virgin rats was significantly less than that of younger animals at 3 and 6 h post feeding but did not differ statistically at the later time intervals. The possibility exists that neoplastic transformation may require the interaction between high levels of DMBA and the constitutents of the mammary parenchymal cells for extended periods of time. Therefore, the decreased exposure of the cellular constituents to DMBA could account for the decrease in mammary cancer incidence observed in older rats.


*Br. J. Cancer* (1975) **31**, 189

### EFFECT OF AGE AND PARITY UPON THE UPTAKE OF 9,10-DIMETHYL-1,2-BENZANTHRACENE-9-$^{14}$C BY MAMMARY PARENCHYMAL CELLS OF THE RAT

C. J. GRUBBS* AND R. C. MOON†

*From the Department of Physiology and Biophysics, University of Tennessee Center for The Health Sciences, Memphis, Tennessee* 38163

Received 28 August 1974. Accepted 17 October 1974

**Summary.—The radioactivity of the parenchymal cell intracellular lipid obtained from 200-day old multiparous animals was significantly less than that of both 50- and 200-day old virgin rats at all time intervals. Furthermore, the parenchymal cell dry, fat-free tissue of the multiparous animals contained significantly less DMBA-9-$^{14}$C than this fraction obtained from young or old virgin rats. Since there was a decrease in both the uptake and binding of DMBA-9-$^{14}$C by the mammary parenchymal cells of multiparous animals, it would appear that factors associated with pregnancy and/or lactation result in an altered susceptibility of the parenchymal cell to this carcinogen.**

**Binding of DMBA-9-$^{14}$C by parenchymal cells of old virgin rats was significantly less than that of younger animals at 3 and 6 h post feeding but did not differ statistically at the later time intervals. The possibility exists that neoplastic transformation may require the interaction between high levels of DMBA and the constituents of the mammary parenchymal cells for extended periods of time. Therefore, the decreased exposure of the cellular constituents to DMBA could account for the decrease in mammary cancer incidence observed in older rats.**

It is generally accepted that certain polycyclic hydrocarbons are extremely effective in inducing mammary cancer in experimental animals. However, variations in the age (Dao, 1969), endocrine status (Huggins, Grand and Brillantes, 1961; Huggins, Moon and Morii, 1962), reproductive history (Mirra, Cole and MacMahon, 1971) and lineage of the animals (Sydnor *et al.*, 1962) will greatly affect the incidence and number of mammary cancers produced by these chemical carcinogens. Moon, Janss and Young (1969) have developed a technique that permits the effective separation of isolated mammary parenchymal cells from mammary adipose cells. Using this method, Janss and Moon (1970) have demonstrated that the concentration of 9,10-dimethyl-1,2-benzanthracene (DMBA) in mammary fat cells obscures the uptake of the carcinogen by the parenchymal cells because of the lipid solubility of the compound. It was further shown that the parenchymal cell intracellular lipid is of prime importance in carcinogen uptake by the parenchymal cell and that DMBA released from this fraction is bound to cellular proteins and DNA (Janss, Moon and Irving, 1972).

Moon (1969) has also demonstrated that rats fed DMBA after having undergone 2 pregnancies and lactations exhibited an incidence of mammary cancer which was significantly less than that of virgin animals of the same age. As a result of these studies, it was suggested that the fluctuations in hormone secretion

* To whom requests for reprints should be addressed, at the Department of Biochemistry, University of Tennessee Center for the Health Sciences, 800 Madison Avenue, Memphis, Tennessee 38163.

† Present Address: IIT Research Institute, 10 West 35th Street, Chicago, Illinois 60616.

concomitant with gestation and/or lactation may result in a decrease in the susceptibility of the mammary parenchymal cells to the carcinogen. Thus, an investigation of the uptake and binding of DMBA-9-$^{14}$C by mammary parenchymal cells from virgin and multiparous rats at different ages appeared warranted.

### MATERIALS AND METHODS

Virgin, female, Sprague–Dawley rats were obtained from Holtzman Company (Madison, Wisconsin) at 40 days of age and were kept in a room artificially lighted 14 h each day and maintained at a temperature of $75 \pm 2$°F. Purina Lab Chow and tap water were given *ad libitum.* Female rats which were to undergo pregnancy and lactation were mated with male Sprague–Dawley rats at 50 days of age. Pregnant animals were placed in individual cages to deliver and remained in such cages during the lactation period. Following parturition, each litter was adjusted to 6 pups and the pups allowed to nurse for 21 days. The dams were isolated for 14 days following the 21-day lactation period to permit involution of the mammary glands before remating. Similar procedures were followed during the second pregnancy and lactational period. Dams which failed to maintain their young for an entire lactation period were not included in these data.

At 200 days of age (approximately 40 days after the last lactation), the multiparous rats received 20 mg of DMBA and 25 $\mu$Ci of DMBA-9-$^{14}$C in 1 ml sesame oil by intragastric instillation. A group of 200-day old virgin animals, as well as a group of 50-day old virgin animals, received the same dose of the labelled and non-labelled DMBA. Crystalline DMBA and DMBA-9-$^{14}$C (S. A., 6·7 mCi/mmol) were obtained from Eastman Kodak Company and Amersham/Searle Corporation, respectively. The purity of both the labelled and non-labelled compounds was determined by thin layer chromatography using Skellysolve B-benzene (50 : 50, v/v) as the solvent system.

The rats of each group were anaesthetized with ether and blood samples were withdrawn from the inferior vena cava at 3, 6, 16, and 24 h after feeding the carcinogen. The animals were sacrified at the various time intervals by an overdose of ether and the right and left abdominal–inguinal mammary glands were rapidly excised and frozen. The glands on the right side of the animal were used for determination of total lipid and DNA content of the entire mammary gland. The glands on the left side of the animal were treated with collagenase (Worthington Biochemical Corp., Code: CLS, Freehold, New Jersey) to yield the mammary parenchymal cell and fat cell fractions (Moon *et al.*, 1969). The isolated mammary parenchymal cell fraction was further separated into the intracellular lipid and dry, fat-free fractions by extracting the intact parenchymal cells with chloroform–methanol (2 : 1, v/v). Total lipid and DNA content were also determined on both the mammary parenchymal and fat cell fractions. Total lipid was extracted by the method of Folch, Lees and Sloane Stanley (1957), while the procedure previously described by Moon (1961) was used to determine mammary DNA content.

Ligatures were placed just above the entrance of the oesophagus into the stomach and above the rectum to prevent the loss of any of the gastrointestinal tract contents. This section of the tract was then removed and trimmed of all adhering adipose tissue. All tissues were weighed, digested with 0·5 N NaOH and counted for radioactivity according to the procedure of Janss and Moon (1970). Samples were counted for 20 min and actual counts for all samples were at least 5 times greater than background. An analysis of variance was performed for the difference between means and any such differences were considered to be statistically significant if the level of confidence was 95% or greater.

### RESULTS

### *Radioactivity*

The patterns of DMBA-9-$^{14}$C uptake by the mammary parenchymal cell intracellular lipid from the 3 groups of animals studied are depicted in Fig. 1. The uptake of DMBA-9-$^{14}$C by the intracellular lipid fraction of 50-day old virgin, 200-day old virgin and 200-day old parous rats reached a maximum value 6 h after the carcinogen was fed and declined slowly over the subsequent 18 h. The radioactivity of this fraction obtained from 200-day old virgin rats was signifi-

FIG. 1.—Comparison of the uptake of DMBA-9-$^{14}$C by mammary gland parenchymal cell intracellular lipid obtained from 50-day old virgin, 200-day old virgin and 200-day old multiparous animals. The rats received 25 $\mu$Ci DMBA-9-$^{14}$C and 20 mg DMBA in 1 ml sesame oil by gastric instillation and were killed at different intervals after feeding. Each bar represents the mean specific activity $\pm$ s.e. (DPM/MG) of at least 5 different animals from which the fraction of collagenase treated mammary gland was obtained.

cantly greater than that of 200-day old parous animals at 3($P < 0{\cdot}05$), 6($P < 0{\cdot}01$), 16($P < 0{\cdot}05$), and 24($P < 0{\cdot}01$) h after DMBA-9-$^{14}$C feeding. Similar results were observed when the radioactivity of the intracellular lipid fraction obtained from 50-day old virgin rats was compared with the same fraction obtained from 200-day old parous animals, although the differences were greater at each time interval.

The radioactivity of the parenchymal cell dry, fat-free tissue (DFFT) obtained from 50-day old virgin, 200-day old virgin and 200-day old multiparous rats is compared in Fig. 2. The pattern of DMBA-9-$^{14}$C uptake by this fraction in the 3 groups was similar, *i.e.*, a gradual increase in binding of the carcinogen at the time intervals observed. The radioactivity of the DFFT of parenchymal cells from 50-day old virgin animals was greater than that of 200-day old parous rats at all periods after the instillation of DMBA-9-$^{14}$C. However, the binding of DMBA to the parenchymal DFFT of 50-day old virgin animals was greater than that of the same fraction from 200-day old virgin rats only at 3($P<0{\cdot}01$) and 6($P < 0{\cdot}05$) h post DMBA-9-$^{14}$C feeding. At 3 and 6 h after the administration of DMBA-9-$^{14}$C, the difference in the radioactivity of the DFFT obtained from 200-day old virgin and parous rats

FIG. 2.—Comparison of the uptake of DMBA-9-$^{14}C$ by mammary gland parenchymal cell dry, fat-free tissue (DFFT) obtained from 50-day old virgin, 200-day old virgin and 200-day old multiparous animals. Experimental protocol was the same as that described in the legend of Fig. 1.

was statistically insignificant. At the later time intervals, however, the uptake of carcinogen by 200-day old virgin animals exceeded that of 200-day old parous rats (16 h, $P < 0{\cdot}05$; 24 h, $P < 0{\cdot}01$).

The radioactivity of the gastrointestinal tract and plasma from the 3 groups of animals at the various time intervals were similar. Thus, it would appear that the absorption and transport of the carcinogen are not altered by the age or reproductive history of the rat.

*Total lipid and DNA content*

Since the wet weight of the intact mammary glands obtained from 200-day old virgin and parous animals was slightly greater than those from 50-day old virgin animals (Table), the lipid content was expressed as a percentage of the wet weight of the mammary gland. The intact tissue from both groups of 200-day old rats contained a greater percent lipid than that of 50-day old virgin animals. The difference in percent total lipid between 200-day old virgin and parous rats was statistically insignificant. The total DNA content of the intact glands from 50-day old animals was less ($P < 0{\cdot}01$) than that of intact glands from 200-day old virgin and parous rats. Because Moon, Griffith and Turner (1959) have shown that a more reliable index of parenchymal cell proliferation is obtained when total DNA is expressed per unit body weight, the total DNA content of mammary glands obtained from the 3 groups was compared on the basis of DNA per 100 g body weight. The data expressed in this manner showed a greater mammary

TABLE.—*Total Lipid and DNA Content of Untreated and Collagenase Treated Mammary Tissue from 50-day Old Virgin, 200-day Old Virgin, and 200-day Old Multiparous Rats*

| Mammary fraction | Mammary wet weight (g) | Percent total lipid[a] | Total DNA (μg) | DNA (μg) / 100 g BW[b] | Cellular ratio[c] |
|---|---|---|---|---|---|
| *50-day old virgin* | | | | | |
| Untreated | 1·42 | 46·7 | 1679·6 | 952·6 | 1·8 |
| | ±0·05[d] | ±2·4 | ±82·2 | ±43·3 | |
| Collagenase treated | 0·10 | 1·8 | 133·6 | 78·6 | |
| parenchymal cells | ±0·02 | ±0·2 | ±12·6 | ±7·4 | |
| Fat cells | 0·45 | 92·4 | 75·9 | 44·6 | |
| | ±0·02 | ±3·1 | ±14·3 | ±8·2 | |
| *200-day old virgin* | | | | | |
| Untreated | 2·00 | 57·4 | 2102·1 | 632·4 | 1·2 |
| | ±0·15 | ±2·6 | ±93·9 | ±22·3 | |
| Collagenase treated | 0·16 | 1·2 | 168·9 | 51·2 | |
| parenchymal cells | ±0·02 | ±0·3 | ±28·8 | ±8·7 | |
| Fat cells | 1·13 | 86·4 | 142·2 | 43·1 | |
| | ±0·08 | ±1·4 | ±14·0 | ±4·3 | |
| *200-day old multiparous* | | | | | |
| Untreated | 1·65 | 55·4 | 2225·4 | 651·8 | 1·0 |
| | ±0·05 | ±1·9 | ±116·2 | ±34·8 | |
| Collagenase treated | 0·16 | 1·5 | 157·9 | 46·4 | |
| parenchymal cells | ±0·02 | ±0·3 | ±10·8 | ±3·2 | |
| Fat cells | 0·84 | 87·4 | 153·6 | 45·3 | |
| | ±0·06 | ±5·6 | ±24·8 | ±9·4 | |

[a] G of total lipid per g of tissue times 100.
[b] μg of DNA per 100 g of body weight.
[c] Parenchymal cell DNA per 100 g body weight divided by fat cell DNA per 100 g body weight.
[d] All values are means ± s.e. of at least 5 animals. Mean body weights of the 50-day old virgin, 200-day old virgin and 200-day old multiparous rats were 170·0 ± 3·5, 332·8 ± 3·7 and 342·5 ± 3·3 respectively.

DNA content for 50-day old virgin rats than for either 200-day old groups.

Analysis of the isolated mammary parenchymal cells for percent intracellular lipid revealed a greater lipid content in cells from 50-day old virgin rats than from 200-day old virgin animals. The mammary fat cells from the 3 groups were composed of approximately 90% lipid as opposed to parenchymal cell intracellular lipid contents of 1·2 : 1·8%. The parenchymal cell to fat cell ratio of 50-day old virgin animals was 1·8 : 1, while this ratio in 200-day old virgin rats was 1·2 : 1 and in 200-day old parous animals 1 : 1.

### DISCUSSION

Huggins *et al.* (1961) were the first to demonstrate the importance of age at the time of carcinogen feeding on the chemical induction of mammary cancer. Although the incidence of mammary cancer is 100% in animals receiving methylcholanthrene (MCA) at 50 days of age, a progressive decline in the induction of mammary tumours occurs if this compound is fed to older rats. Moon (1969) found a mammary cancer incidence of 39% in rats receiving DMBA at 190 days of age. Similar results were obtained by Meranze, Gruenstein and Shimkin (1969) in rats receiving this carcinogen at 6 to 7 months of age and by Dao (1969) in which mammary glands from young and old donors exposed to DMBA were transplanted to untreated isologous recipients.

In the present study, the concentration of DMBA was greater in the parenchymal cell intracellular lipid of 50-day old virgin rats than in 200-day old virgin animals at all time intervals, although significant differences were observed only at 3 and 16 h after feeding the carcinogen. An analysis of the percent intracellular lipid revealed a greater lipid content in mammary parenchymal cells of 50-day old rats than that observed in the 200-day old virgin animals. Since DMBA is lipophilic, this could partially explain the higher radioactivity found in the parenchymal cell intracellular lipid of the younger rats. However, since the metabolic activity of mammary parenchymal cells from young or old rats has not been determined, a difference in lipid composition or transport of the carcinogen within the mammary parenchymal cell cannot be eliminated.

Huggins *et al.* (1961) have demonstrated that the optimal single oral dose of MCA for the induction of mammary cancer in the rat is 100 mg whereas the optimal dose of DMBA under the same conditions is 20 mg. If smaller doses of these polycyclic hydrocarbons are administered, the incidence of mammary cancer decreases, the time of tumour appearance increases and the number of tumours per rat diminishes. Hence, it is apparent that a critical level of DMBA must reach the mammary parenchymal cells in order to induce neoplastic alteration. Janss and Moon (1970) have shown that maximum binding to the parenchymal cell dry, fat-free residue occurs at 16–24 h post-feeding of DMBA. A later study (Janss *et al.*, 1972) confirmed that maximum binding to DNA also occurred during the first day after DMBA administration. Furthermore, Janss and Moon (1971) found that less DMBA was bound to the parenchymal cells of ovariectomized and hypophysectomized rats than intact animals during the first 24 h after intragastric feeding. Therefore, the extent of binding (a function of time and quantity) to the parenchymal cellular constituents would appear to be a determining factor for neoplastic transformation.

The present data indicated that the concentration of DMBA in the DFFT of 200-day old virgin animals was similar to that of 50-day old rats at 16 and 24 h after feeding. However, at the earlier time periods, the binding of DMBA to cellular constituents was considerably less in the older animals. Such a difference in binding would alter the time of exposure of the cells to high levels of DMBA and could account for the decrease in incidence of mammary tumours when this carcinogen is administered to older rats.

The frequency of breast cancer in the human is greater in nulliparous women than in those having undergone pregnancy (Logan, 1953; Wynder, Bross and Hirayama, 1960; Sydnor *et al.*, 1962). Several studies (Mirra *et al.*, 1971; Salber, Trichopoulas and MacMahon, 1969; Yuasa and MacMahon, 1970) however, have suggested that the age of the mother at the first pregnancy may be of more importance than the number of pregnancies in decreasing the incidence of breast cancer in multiparous women. An inverse relationship has also been demonstrated between parity and DMBA induced mammary cancer in rats (Moon, 1969). Animals having undergone 2 pregnancies before receiving DMBA exhibited a significant decrease in the incidence of mammary cancer relative to virgin control rats of the same age. It was suggested that the fluctuations in ovarian and pituitary hormone secretion which occur in parous rats might result in either a reduction of rat mammary parenchymal cell susceptibility to the carcinogen or a decrease in neoplastic cell sensitivity to hormones which influence tumour growth.

The radioactivity of the parenchymal cell intracellular lipid of 200-day old multiparous animals was significantly less than that observed in young and old virgin rats at all time intervals. The lipid content of the mammary parenchymal cells obtained from parous rats was not statistically different from the intracellular lipid content of either 50- or 200-day old virgin animals. Although binding of DMBA in the multiparous rats increased at each time period after feeding of the carcinogen, the radioactivity of the parenchymal cell DFFT was less than that of young virgin rats.

Thus, the decrease in binding of DMBA to the parenchymal cell non-lipid residue of parous rats is probably a reflection of the smaller uptake of carcinogen by the intracellular lipid.

These studies on the interaction of DMBA with mammary parenchymal cells demonstrate that age and parity will indeed alter the uptake and binding of this carcinogen by the parenchymal cell. Factors associated with pregnancy and/or lactation resulted in a decrease in the binding of DMBA to the mammary parenchymal cells at all time intervals during the first 24 h after carcinogen instillation. Although the level of binding of DMBA to mammary parenchymal cells of 200-day old virgin animals approached that of 50-day old virgin rats at 16 to 24 h post feeding, the time of exposure of the parenchymal cell DNA, RNA and proteins to these high levels of carcinogen was shortened. Janss and Ben (1974) found that binding of DMBA to mammary gland DNA from rats 35 days of age (which exhibit a greatly reduced tumour incidence) was 40–50% less than that of animals receiving DMBA at 50 days of age. The present data suggest that binding to DNA of 200-day old animals may also be reduced and experiments are currently planned to investigate this further.

Since the 3 groups of rats received the same dose (20 mg) of DMBA, the possibility exists that part of the differences observed between 50- and 200-day old animals was due to differences in body weight. However, since no differences were observed in plasma concentration of DMBA-9-$^{14}$C in the 3 groups of animals, it is apparent that the mammary glands were exposed to similar quantities of the carcinogen. Therefore, the lower incidence of mammary cancer following the feeding of DMBA to older virgin or multiparous rats is most likely the result of a decrease in the susceptibility of the mammary parenchymal cells to the carcinogen as suggested earlier by Moon (1969).

The excellent technical assistance of Mrs Karen Gustafson and Mrs Mary Smith is greatly appreciated. This research was supported by grants from Jane Coffin Childs Memorial Fund No. 221, Milheim Foundation for Cancer Research No. 69-12, USPHS Grant CA-12630, and ACS Grant IN-85-E7.

### REFERENCES

Dao, T. L. (1969) Mammary Cancer Induction by 7,12-Dimethylbenz(a)anthracene: Relation to Age. *Science, N.Y.*, **165**, 810.

Folch, J., Lees, M. & Sloane Stanley, G. H. (1957) A Simple Method for the Isolation and Purification of Total Lipids from Animal Tissues. *J. biol. Chem.*, **226**, 497.

Huggins, C., Grand, L. C. & Brillantes, F. P. (1961) Mammary Cancer Induced by a Single Feeding of Polynuclear Hydrocarbons, and Its Suppression. *Nature, Lond.*, **189**, 204.

Huggins, C., Moon, R. C. & Morii, S. (1962) Extinction of Experimental Mammary Cancer, I. Estradiol-17$\beta$ and Progesterone. *Proc. natn. Acad. Sci. U.S.A.*, **48**, 379.

Janss, D. H. & Ben, T. L. (1974) Influence of Age on the Binding of 7,12-Dimethylbenz(a)anthracene (DMBA) to Rat Mammary Epithelial Cell DNA *in vivo*. *Proc. Am. As. Cancer Res.*, **15**, 120.

Janss, D. H. & Moon, R. C. (1970) Uptake and Clearance of 9,10-Dimethyl-1,2-benzenthracene-9-$^{14}$C by Mammary Parenchymal Cells of the Rat. *Cancer Res.*, **30**, 473.

Janss, D. H. & Moon, R. C. (1971) Effect of Endocrine Organ Ablation on the Uptake and Clearance of 9,10-Dimethyl-1,2-benzanthracene-9-$^{14}$C by Mammary Parenchymal Cells of the Rat. *Cancer Res.*, **31**, 2026.

Janss, D. H., Moon, R. C. & Irving, C. C. (1972) The Binding of 7,12-Dimethylbenz(a)anthracene to Mammary Parenchymal DNA and Protein *in vivo*. *Cancer Res.*, **32**, 254.

Logan, W. P. D. (1953) Marriage and Childbearing in Relation to Cancer of the Breast and Uterus. *Lancet*, ii, 1199.

Meranze, D. R., Gruenstein, M. & Shimkin, M. B. (1969) Effect of Age and Sex on the Development of Neoplasms in Wistar Rats Receiving a Single Intragastric Instillation of 7,12-Dimethylbenz(a)-anthracene. *Int. J. Cancer*, **4**, 480.

Mirra, A. P., Cole, P. & MacMahon, B. (1971) Breast Cancer in an Area of High Parity: Sao Paulo, Brazil. *Cancer Res.*, **31**, 77.

Moon, R. C. (1961) Growth Hormone and Mammary Gland Lobule-Alveolar Development. *Am. J. Physiol.*, **201**, 259.

Moon, R. C. (1969) Relationship Between Previous Reproductive History and Chemically Induced Mammary Cancer in Rats. *Int. J. Cancer*, **4**, 312.

Moon, R. C., Griffith, D. R. & Turner, C. W. (1959) Normal and Experimental Growth of Rat Mammary Gland. *Proc. Soc. exp. Biol. Med.*, **101**, 788.

MOON, R. C., JANSS, D. H. & YOUNG, S. (1969) Preparation of Fat Cell-" Free " Rat Mammary Gland. *J. Histochem. Cytochem.*, **17**, 182.

SALBER, E. J., TRICHOPOULOS, D. & MACMAHON, B. (1969) Lactation and Reproduction Histories of Breast Cancer Patients in Boston, 1965–1966. *J. natn. Cancer Inst.*, **43**, 1013.

SYDNOR, K. L., BUTENANDT, O., BRILLANTES, F. P. & HUGGINS, C. (1962) Race-strain Factor Related to Hydrocarbon-induced Mammary Cancer in Rats. *J. natn. Cancer Inst.*, **29**, 805.

WYNDER, E. L., BROSS, I. J. & HIRAYAMA, T. (1960) A Study of the Epidemiology of Cancer of the Breast. *Cancer, N.Y.*, **13**, 559.

YUASA, S. & MACMAHON, B. (1970) Lactation and Reproductive Histories of Breast Cancer Patients in Tokyo, Japan. *Bull. Wld Hlth Org.*, **42**, 195.